# Conumee 2.0: enhanced copy-number variation analysis from DNA methylation arrays for humans and mice

**DOI:** 10.1093/bioinformatics/btae029

**Published:** 2024-01-19

**Authors:** Bjarne Daenekas, Eilís Pérez, Fabio Boniolo, Sabina Stefan, Salvatore Benfatto, Martin Sill, Dominik Sturm, David T W Jones, David Capper, Marc Zapatka, Volker Hovestadt

**Affiliations:** Department of Pediatric Oncology, Dana-Farber Cancer Institute, Boston, MA 02115, United States; Broad Institute of MIT and Harvard, Cambridge, MA 02142, United States; Department of Neuropathology, Charité – Universitätsmedizin Berlin, Corporate Member of Freie Universität Berlin and Humboldt-Universität zu Berlin, 10117 Berlin, Germany; Department of Neuropathology, Charité – Universitätsmedizin Berlin, Corporate Member of Freie Universität Berlin and Humboldt-Universität zu Berlin, 10117 Berlin, Germany; Department of Pediatric Oncology, Dana-Farber Cancer Institute, Boston, MA 02115, United States; Broad Institute of MIT and Harvard, Cambridge, MA 02142, United States; Department of Pediatric Oncology, Dana-Farber Cancer Institute, Boston, MA 02115, United States; Broad Institute of MIT and Harvard, Cambridge, MA 02142, United States; Department of Pediatric Oncology, Dana-Farber Cancer Institute, Boston, MA 02115, United States; Broad Institute of MIT and Harvard, Cambridge, MA 02142, United States; Hopp Children’s Cancer Center Heidelberg (KiTZ), 69120 Heidelberg, Germany; Division of Pediatric Neurooncology, German Cancer Research Center (DKFZ) and German Cancer Consortium (DKTK), 69120 Heidelberg, Germany; Hopp Children’s Cancer Center Heidelberg (KiTZ), 69120 Heidelberg, Germany; Division of Pediatric Glioma Research, German Cancer Research Center (DKFZ) and German Cancer Consortium (DKTK), 69120 Heidelberg, Germany; Department of Pediatric Oncology, Hematology & Immunology, Heidelberg University Hospital, 69120 Heidelberg, Germany; Hopp Children’s Cancer Center Heidelberg (KiTZ), 69120 Heidelberg, Germany; Division of Pediatric Glioma Research, German Cancer Research Center (DKFZ) and German Cancer Consortium (DKTK), 69120 Heidelberg, Germany; Department of Neuropathology, Charité – Universitätsmedizin Berlin, Corporate Member of Freie Universität Berlin and Humboldt-Universität zu Berlin, 10117 Berlin, Germany; German Cancer Consortium (DKTK), Partner Site Berlin, German Cancer Research Center (DKFZ), 69120 Heidelberg, Germany; Division of Molecular Genetics, German Cancer Research Center (DKFZ), 69120 Heidelberg, Germany; Department of Pediatric Oncology, Dana-Farber Cancer Institute, Boston, MA 02115, United States; Broad Institute of MIT and Harvard, Cambridge, MA 02142, United States

## Abstract

**Motivation:**

Copy-number variations (CNVs) are common genetic alterations in cancer and their detection may impact tumor classification and therapeutic decisions. However, detection of clinically relevant large and focal CNVs remains challenging when sample material or resources are limited. This has motivated us to create a software tool to infer CNVs from DNA methylation arrays which are often generated as part of clinical routines and in research settings.

**Results:**

We present our R package, conumee 2.0, that combines tangent normalization, an adjustable genomic binning heuristic, and weighted circular binary segmentation to utilize DNA methylation arrays for CNV analysis and mitigate technical biases and batch effects. Segmentation results were validated in a lung squamous cell carcinoma dataset from TCGA (*n* = 367 samples) by comparison to segmentations derived from genotyping arrays (Pearson’s correlation coefficient of 0.91). We further introduce a segmented block bootstrapping approach to detect focal alternations that achieved 60.9% sensitivity and 98.6% specificity for deletions affecting *CDKN2A/B* (60.0% and 96.9% for *RB1*, respectively) in a low-grade glioma cohort from TCGA (*n* = 239 samples). Finally, our tool provides functionality to detect and summarize CNVs across large sample cohorts.

**Availability and implementation:**

Conumee 2.0 is available under open-source license at: https://github.com/hovestadtlab/conumee2.

## 1 Introduction

Copy-number variations (CNVs) are genomic segments that exhibit differences in chromosomal copy-number states based on the comparison of two or more genomes (Hastings *et al.* 2009, MacDonald *et al.* 2014). The lengths of these segments vary over orders of magnitude, resulting in a stratification of large (chromosome arm-level gains and losses) and focal (amplifications and deletions) variations. The majority of germline CNVs contribute to the genetic variability among individuals and typically do not imply pathogenic potential. When affecting genes or regulatory elements, germline CNVs may result in gene malfunction or changes in expression levels, manifesting in diseases like autism spectrum disorder and Alzheimer’s disease (Levy *et al.* 2011, Cuccaro *et al.* 2017). Somatically acquired CNVs are of particular relevance for oncogenesis. Cancer forms through the consecutive acquisition of genomic alterations such as point mutations (single nucleotide variants), short insertions and deletions, focal and large-scale CNVs, and epigenetic alterations, including changes in DNA methylation and histone modification patterns (Jones and Baylin 2007, Beroukhim *et al.* 2010). During tumor formation and progression, these alterations confer a selective advantage and are selected in an evolutionary process. Prominent oncogenes and tumor suppressor genes that are affected by CNVs include epidermal growth factor receptor (*EGFR*), retinoblastoma protein (*RB1*), and the cellular tumor antigen p53 (*TP53*), which have pivotal roles in tumorigenesis and are targets in cancer therapy (Ciardiello and Tortora 2008, Lee and Muller 2010). Many other CNVs have been implicated as driver events in various types of cancer. Hence, the ability to accurately identify CNVs is important to unravel pathomechanisms and potential therapeutic vulnerabilities.

Whole-genome next generation sequencing and genotyping microarrays are considered gold standards for CNV analysis, as they provide the most extensive genome coverage (Mariani *et al.* 2022). Another genome-wide assay that is widely applied in biological research and clinical settings are DNA methylation microarrays (i.e. Illumina Infinium BeadChip arrays). Profiling of DNA methylation, a central epigenetic mark, has enabled the discovery and delineation of molecular classes of brain tumors, sarcomas, leukemias, and many other types of cancer (Sturm *et al.* 2016, Capper *et al.* 2018, Giacopelli *et al.* 2021, Kölsche *et al.* 2021). A recent machine learning-based patient classification system for brain tumors (often referred to as the “Heidelberg classifier”) showcases the high clinical utility of this data type and is integrated in routine workflows worldwide, having classified over 100 000 cases as of December 2022 (Capper *et al.* 2018, Sturm *et al.* 2023). We show that, in addition to epigenetic information (DNA methylation profiles), it is possible to extract genetic information (CNV profiles) from DNA methylation microarrays, without the need to run a separate genomic assay. This is especially beneficial when sample material or resources are limited.

We first presented our approach in studies of pediatric high-grade glioma and medulloblastoma (Sturm *et al.* 2012, Hovestadt *et al.* 2013) and made it available to the research community as the conumee Bioconductor package in 2015 (Hovestadt and Zapatka 2015). With nearly 20 000 downloads, conumee is one of the most widely used tools for inferring CNVs from DNA methylation arrays and has been applied in numerous large-scale cancer research projects (Sturm *et al.* 2016, Northcott *et al.* 2017). Conumee-derived CNV profiles are frequently displayed in the most recent WHO classification of Tumors of the Central Nervous System and are integrated in automated molecular reports of the Heidelberg classifier, where inferred CNVs may have an impact on tumor classification and clinical decisions (Capper *et al.* 2018, WHO Classification of Tumours Editorial Board 2021). Other tools for analyzing CNVs from methylation arrays include ChAMP (Feber *et al.* 2014), Epicopy (Cho *et al.* 2019), and cnAnalysis450k (Knoll *et al.* 2017).

Here, we present a substantially enhanced version of our package (conumee 2.0) that enables the identification and annotation of focal alterations that affect individual genes using a new statistical approach. We further implement a revised tangent normalization step that increases the signal-to-noise ratio, and extend compatibility to new array types (human EPIC v2.0 array, mouse 285k array). The update also provides functionality for the simultaneous analysis of multiple query samples, adds new plotting functions to visualize recurrent CNVs as well as user-friendly interactive plots that facilitate the identification of relevant genes, and provides text-based output files that are suitable for downstream visualization or processing in other tools (e.g. GISTIC or the IGV browser). The revised algorithm for noise reduction and the performance of the segmentation algorithm were assessed on a lung squamous carcinoma (LUSC) dataset from The Cancer Genome Atlas (TCGA) that comprise both DNA methylation array data and CNV segmentations derived from SNP arrays for paired samples. The detection of focal high-level alterations was assessed on a low-grade glioma (LGG) cohort from TCGA.

## 2 Materials and methods

### 2.1 Data import and probe annotation

Our tool uses DNA methylation data generated using Illumina’s Infinium HumanMethylation450 BeadChip array that covers >480 000 positions across intra- and intergenic regions of the human genome (Bibikova *et al.* 2011). Its successors, the Infinium MethylationEPIC array (EPIC, >850 000 probes) and MethylationEPIC v2.0 array (EPICv2, >930 000 probes), are also supported. To import datasets from raw IDAT files, conumee offers seamless integration with the popular minfi package (Aryee *et al.* 2014). More recent EPICv2 data are imported using functions from the illuminaio package (Smith *et al.* 2013), as the minfi package currently lacks functionality for these arrays. Mouse arrays are imported using the RnBeads package (Müller *et al.* 2019). Probe annotations are loaded from the IlluminaHumanMethylation450kanno.ilmn12.hg19 and IlluminaHumanMethylationEPICanno.ilm10b4.hg19 packages. For the EPICv2 and mouse arrays, probe annotations were downloaded from the manufacturer’s website and a genomic liftover was performed if necessary. In addition, information such as chromosome sizes, centromere position and gaps in the genome assembly are collected from the UCSC Genome Browser.

### 2.2 Tangent normalization

As the first step of the analysis, conumee sums up the unmethylated (*U*) and methylated (*M*) signal intensities for each probe *i* to obtain the combined signal intensity *I*. This is based on the assumption that the combined signal intensity values from the unmethylated and methylated probes are a proxy of the copy-number status of that locus:

$$I_{i}=M_{i}+U_{i}$$

The summed signal intensities are used to perform tangent normalization (Gao *et al.* 2022). To minimize the effect of technical biases and batch effects that arise due to differences in experimental conditions in a given query sample *q*, we normalize the intensity *I* of each probe *i* with a reference intensity profile consisting of the linear combination of a set of control samples *c*. In the initial version of conumee, we fit a linear model using the raw summed intensity values to identify the relative contribution of each control. In the revised version of conumee, we fit the model to log_2_-transformed signal intensities. The log_2_-ratio *R* of probe intensities of a query sample *q* versus the linear combination of control samples *c* (normalized intensities) is calculated and used for further analysis:

conumee:

$$R_{i}=\;\log_{2}(I_{qi})\;-\;\log_{2}\left(\sum_{c\;=\;1}^{n}a_{c}\;I_{ci}\right)$$

conumee 2.0:

$$R_{i}=\;{\text{log}}_{2}(I_{\text{qi}})-\sum_{c=1}^{n}a_{c}\;\;{\text{log}}_{2}\left(I_{\text{ci}}\right)$$

### 2.3 Genomic binning

After the Tangent Normalization is performed, we use an iterative algorithm to merge individual probes into genomic bins. Binning is performed by splitting the genome into segments of a defined size (50 kb by default). The algorithm then selects bins containing less than a defined number of probes (15 probes by default). Every identified bin is merged with the neighboring bin that has fewer probes until a minimum number of probes and minimum genomic size is achieved, resulting in ∼15 000 genomic bins using default parameters for 450k arrays. The normalized signal intensity for each bin is defined as the median log_2_-ratio of all contained probes. The genomic binning heuristic is independent of copy-number states and hence bins are identical between samples. To perform baseline correction (i.e. determine the copy-number neutral state), original bin-level log_2_-ratios are shifted by a centering factor that results in the smallest median absolute deviation to the baseline.

### 2.4 Circular binary segmentation

Finally, segmentation of bins into regions of the same copy-number is performed by using the circular binary segmentation algorithm (Olshen *et al.* 2004). This algorithm treats the genome like a circle and creates partitions trying to maximize the difference in partial means of the intervals. Once this difference is significant, the interval is marked as a segment and the algorithm is applied recursively to the remaining intervals. Conumee 2.0 implements several functions from the DNAcopy package with optimized, but adjustable parameters (Seshan 2022). Results can be visualized by using different plotting functions including illustrations of the whole genome, specified chromosomes or predefined regions of special interest. Recurrent CNVs within a set of query samples can be visualized in summary genome plots. Segments from all analyzed query samples are converted into non-overlapping, referential segments and the type of alteration (gain, loss or balanced) are summarized and visualized as percentages. The thresholds that are used for this summarization step are in line with default parameters used in GISTIC but can be adjusted by the user (Mermel *et al.* 2011). We use the plotly package to generate interactive plots (Sievert 2020).

### 2.5 Segmented block bootstrapping

To calculate empirical *P*-values for focal alterations, we generate random bootstraps of the original dataset by sampling large blocks of bins (block length of 500 kb, 100 bootstrap iterations). Only blocks of bins that are assigned to the same copy-number state (deletions, balanced segments, gains) are concatenated in each bootstrap iteration by using the bootRanges function from the nullranges package (Mu *et al.* 2023). To assign a state to each bin, we perform a k-means clustering (centers = 3). The bootstrapped dataset is used to define two-sided confidence intervals and sample-specific log_2_-ratio thresholds for deletions and amplifications. Subsequently, we determine the log_2_-ratio value of every gene by calculating the median normalized intensity of overlapping probes. Significant genes are identified by applying the dynamic thresholds derived from segmented block bootstrapping. Genes are then overlapped with predefined genes-of-interest and a list of over 700 common onco- and tumor suppressor genes from the Cancer Gene Census (Tate *et al.* 2018).

### 2.6 Validation and benchmarking

Lung squamous carcinoma (LUSC) samples from the Cancer Genome Atlas (TCGA) were chosen to measure the effect of noise reduction as they exhibit abundant CNVs (Cancer Genome Atlas Research Network 2012, Steele *et al.* 2022). The cohort (*n* = 367) comprises matching Illumina 450k and Affymetrix SNP6 array data which was used to validate the performance of our CNV calling algorithm in humans. The raw IDAT files (TCGA level 1 data) were downloaded from the TCGA public repository including methylation profiles of 42 healthy control samples. The segmentation files from the Affymetrix SNP6 arrays (TCGA level 3 data, downloaded in September 2022) were downloaded from Broad Institute’s Firehose Genome Data Analysis Center (data analysis version: 2016_01_28). The GISTIC results for single genes inferred from Affymetrix SNP6 arrays were obtained using the TCGAbiolinks package (Colaprico *et al.* 2015).

To validate our segmented bootstrapping approach for detecting focal high-level alterations, we analyzed the TCGA low grade glioma (LGG) cohort comprising 239 samples with paired SNP array (Affymetrix SNP 6.0) and methylation array (Illumina 450k) data (Brat *et al.* 2015). The raw IDAT files were downloaded from the TCGA public repository (TCGA, level 1 data, January 2023). The 53 reference samples that were used for the LGG cohort were downloaded from GEO (GSE109381, Supplementary Table S1). The copy-number states for *CDKN2A/B* and *RB1* inferred from the Affymetrix SNP6 arrays were downloaded using the TCGAbiolinks package (January 2023) (Colaprico *et al.* 2015).

To quantify the effect of noise reduction in our revised tangent normalization, we define the noise parameter as the average difference in normalized signal intensities of neighboring probes:

$$\begin{array}{c} n\mathrm{oise}=\;\sqrt{\frac{\sum_{i=1}^{n-1}{\left(R_{i+1}-\;R_{i}\right)}^{2}}{n-1}}\\ i=\;\left\{1,2,3\ldots n\right\} \end{array}$$

We validated our segmentation results on the gene-level. The SNP array data served as a reference. Presumably, the borders of the segments are not restricted to gene locations which causes some genes to span multiple segments, especially in the segmentation results from the SNP arrays. To address this, we calculated the gene-wise weighted mean of overlapping segments’ log_2_-ratios that takes the length of the intersection into account. Subsequently, we performed pairwise correlation analysis between the DNA methylation array dataset and SNP arrays dataset and calculated the mean Pearson’s correlation coefficient for every pairwise comparison. To evaluate segmentation calls (i.e. gains and losses) that are above/below a threshold value, we created confusion matrices to evaluate the sensitivity and specificity over all genes across samples.

The GISTIC 2.0 analysis for the segmentation results from both array types was performed using the online platform GenePattern (Reich *et al.* 2006) with the following parameters: amplification threshold = 0.1, deletion threshold = 0.1, cap values = 1.5, broad length cutoff = 0.7, remove X-chromosome = 0, confidence level = 0.99, join segment size = 4, arm level peel off = 1, maximum sample segments = 2000, gene GISTIC = 1. The CNV analysis using ChAMP and cnAnalysis450k was performed with default parameters following the package’s vignette. For comparison of focal CNV detection, we obtained dynamic thresholds from conumee K_CN_ by following the author’s instructions on their github repository.

## 3 Results

### 3.1 Workflow overview

**Figure 1.**
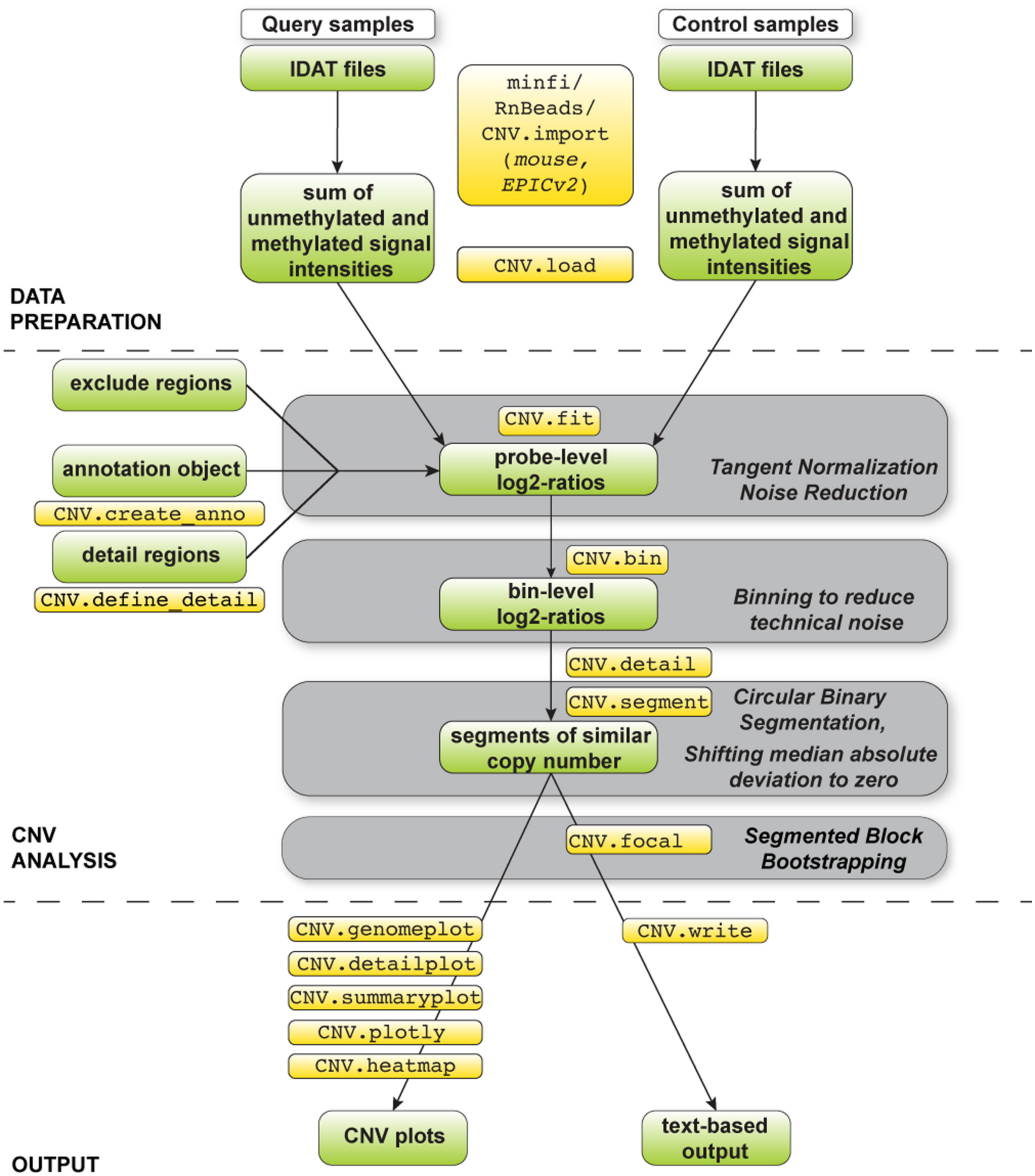
Illustration of the conumee 2.0 workflow. Data objects and commands are indicated. Gray boxes illustrate key steps of the workflow. During data preparation, summed signal intensities for query and control samples are calculated separately. The CNV analysis itself comprises tangent normalization, a genomic binning step, circular binary segmentation, and segmented block bootstrapping.

Extracting information about CNVs from DNA methylation arrays is based on the assumption that the sum of the intensity values of the unmethylated and methylated signal are representative of the copy-number state of a given locus (Fig. 1, top). Our approach follows a three-step workflow comprising data preparation, data analysis, and output generation. We first perform tangent normalization of intensity values from a query sample to determine a unique linear combination of copy-number neutral control samples in order to reduce technical noise (Fig. 1, middle) (Gao *et al.* 2022). As has been shown for CNV analysis from genotyping arrays, the linear combination of control samples approximates the noise profile of a given query sample better than any individual control sample. After calculating the log_2_-ratio of observed (query sample) and fitted (combined control samples) values for each probe, we employ an adaptable heuristic to merge neighboring probes into genomic bins to further reduce technical variability. For each bin, the median of the normalized summed intensity values is calculated and subjected to baseline correction. Large-scale CNVs are detected from genomic bins (log_2_-ratio values) using the circular binary segmentation algorithm (Olshen *et al.* 2004). Focal CNVs, such as high-level amplifications and homozygous deletions, are detected using a novel block bootstrapping approach (Mu *et al.* 2023). Finally, our method provides functionality to produce publication-grade visualizations of CNVs across the genome, selected chromosomes, and individual genes, as well as text-based outputs for downstream processing (Fig. 1, bottom).

### 3.2 Revised CNV calling algorithm

**Figure 2.**
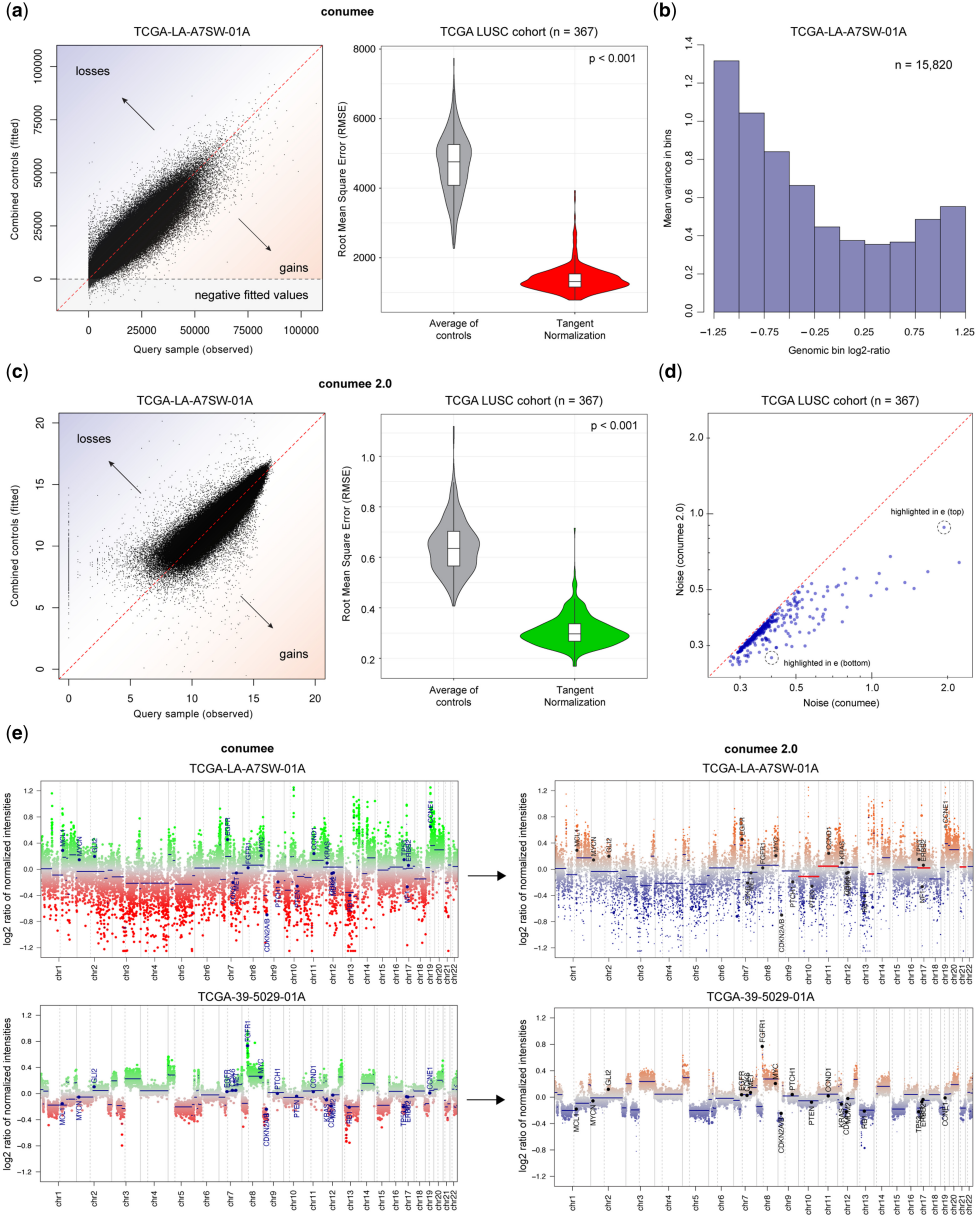
Enhanced conumee algorithm. (a) Scatter plot shows a linear combination of a set of control samples that is fitted against a single query sample in the original tangent normalization approach. Violin plot shows the root mean square error (RMSE) for a set of 367 samples from the TCGA LUSC cohort, using the mean intensity across control samples or the tangent normalization approach. (b) Barplot shows the average probe variance within genomic bins. Higher probe variance is observed in low-intensity bins. (c) Scatter plot shows the updated tangent normalization approach implemented in conumee 2.0 that uses signal intensities that have been log_2_-transformed. Violin plot shows RMSE values using the mean intensity across control samples or the updated tangent normalization approach. (d) Scatter plots shows the noise level for the original (*x*-axis) and the updated (*y*-axis) tangent normalization approach for the TCGA LUSC cohort. The updated approach substantially reduces the noise in many samples. (e) Genome plots show results from the original (left) and updated (right) version of conumee for a low quality (top) and a high quality (bottom) sample. Weighted circular binary segmentation enables a more harmonic segmentation in low quality samples (major segmentation differences are highlighted in red). Weights are visualized as varying dot sizes representing individual bins.

A key step of our approach is the tangent normalization of summed intensity values using a reference of copy-number neutral control samples. Tangent normalization assumes that technical noise present in a given query sample can be approximated by fitting a unique linear combination of copy-number neutral control samples in which technical noise is also present. The fitted noise profile is then subtracted from the query sample (log_2_-ratios). In the previous version of conumee, tangent normalization was performed on untransformed (raw) intensity values. Across 367 tumor samples from the TCGA LUSC project, using 42 controls from the same project as a reference, intensity values from query samples (observed) were much better approximated (fitted) using this approach, compared to using the average intensity of control samples (*P* < .001, Wilcoxon signed-rank test; Fig. 2a, right). Notably, this step reduces the probe-level technical noise for each sample, but is not expected to reduce changes in signal intensities resulting from CNVs.

When investigating the contribution of individual control samples to the tangent normalization, we observed that a unique combination of nearly all samples was used for the fit (Supplementary Fig. S1). Frequently, select control samples contributed more prominently than others, and some were associated with negative coefficients. We also observed that a fraction of fitted control intensities were negative, which was especially pronounced for low-quality query samples that contain many low intensity probes (Fig. 2a). During the calculation of bin-level log_2_-ratios, these negative control intensities were set to 1. We found that the problematic fitting of low signal query intensities resulted in a higher variance within bins that were associated with overall low or high log_2_-ratios (Fig. 2b).

In the enhanced version of conumee, tangent normalization is performed on log_2_-transformed summed intensity values (Fig. 2c). The revised tangent normalization again achieved a higher concordance compared to taking the average intensity of control samples. Probes with low signal intensities are now fitted more truthfully (Supplementary Fig. S2). For a direct comparison of the original and revised tangent normalization approach, we quantified the average difference in normalized intensities between all neighboring probes in a given profile, reasoning that probes in close proximity are likely to be associated with the same underlying copy-number state and differences are representing technical noise. Conumee 2.0 achieved a significantly lower noise parameter in every sample of the cohort (*P* < .001, Wilcoxon signed-rank test; Fig. 2d).

Motivated by the higher probe variance in bins that were associated with more extreme log_2_-ratios, we further optimized the revised version of conumee by implementing a weighted circular binary segmentation approach. Segmentation is performed on genomic bins, which contain at least 15 individual probes (default settings). We assign a weight to each bin that is inverse to the variance of normalized probe intensities, thereby reducing the influence of bins that are associated with a higher probe variance. This leads to differences in the segmentation output, especially in lower quality samples (Fig. 2e).

### 3.3 Validation of CNV results

**Figure 3.**
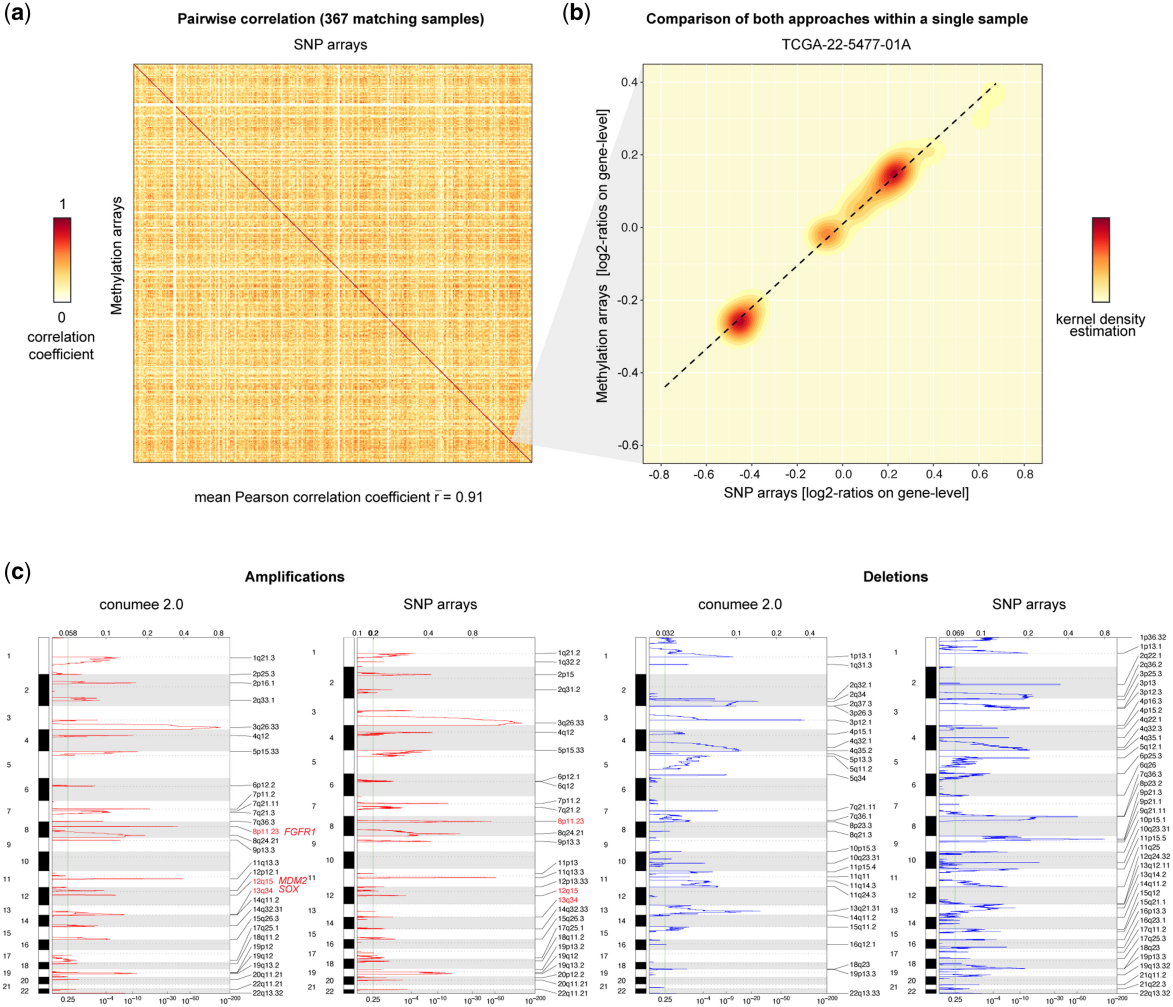
Performance of the revised approach. (a) Heatmap shows the pairwise correlation between SNP array-derived (*x*-axis) and DNA methylation array-derived (*y*-axis) CNVs across all 367 TCGA LUSC tumors. Highest correlation coefficients are observed for data generated from the same tumor on the diagonal. (b) Density heatmap (kernel density estimation) shows the correlation between both array types for a single query. (c) qplots generated using GISTIC 2.0 from conumee 2.0 output illustrate recurrent amplifications (left) and deletions (right), as analyzed from DNA methylation and SNP arrays. Known hallmark alterations of LUSC are indicated in red.

To assess the accuracy of resulting segmentations, we compared DNA methylation array-derived CNVs (Illumina 450k) to SNP array-derived CNVs (Affymetrix SNP 6.0) from the TCGA LUSC patient cohort (Fig. 3a). Highest correlation between segmentation results from both data types was observed for matching samples, achieving an average Pearson’s correlation coefficient of 0.91 (standard deviation: 0.14). Non-matching samples, which may harbor different CNVs, were associated with an average correlation coefficient of 0.3 (standard deviation: 0.14). In comparison, performing CNV segmentation on the same dataset using the ChAMP or cnAnalysis450k packages resulted in lower average correlation coefficients for matching samples (Supplementary Fig. S3). At the level of individual samples, we could confirm a linear correlation between the SNP and methylation array data (Fig. 3b). Notably, the absolute values from the methylation data were often smaller than from the SNP array data. Using fixed thresholds to obtain binary CNV calls (i.e. genomic segments that gained or lost), we achieved a median sensitivity of 88.5% and 91.1% and a median specificity of 98.3% and 97.7% for gains and losses, respectively (Supplementary Figs S4 and S5).

The updated conumee package also provides text-based outputs of resulting segmentations. These files enable compatibility with GISTIC 2.0, a popular tool that uses an advanced probabilistic method to identify recurrent CNVs with potential biological relevance in a set of query samples (Mermel *et al.* 2011). We performed GISTIC analysis on DNA methylation-derived and SNP array-derived segmentations from the TCGA LUSC cohort (Fig. 3c). This comparison demonstrates that most known hallmark amplifications in lung squamous carcinoma, including *FGFR1* (8p11.23), *SOX1* (13q34) and *MDM2* (12q15), are detected by conumee 2.0. Some focal deletions, such as those on the short arms of chromosome 8 and 9, are missed.

### 3.4 Gene-level analysis and detection of focal alterations

There is a strong need to accurately report clinically relevant focal CNVs, including high-level amplifications and homozygous deletions of genes that are part of diagnostic criteria for certain cancer entities (WHO Classification of Tumours Editorial Board 2021). Clinicians and researchers often rely on fixed thresholds to detect these alterations. This approach does not take into account that optimal thresholds may vary for different genes and/or pathologies (e.g. distinct cancer entities), and are dependent on tumor purity and data quality. Recent work by Blecua *et al.* addresses this challenge by adapting conumee to include dynamic sample-dependent thresholds that take tumor purity into account (Blecua *et al.* 2022).

**Figure 4.**
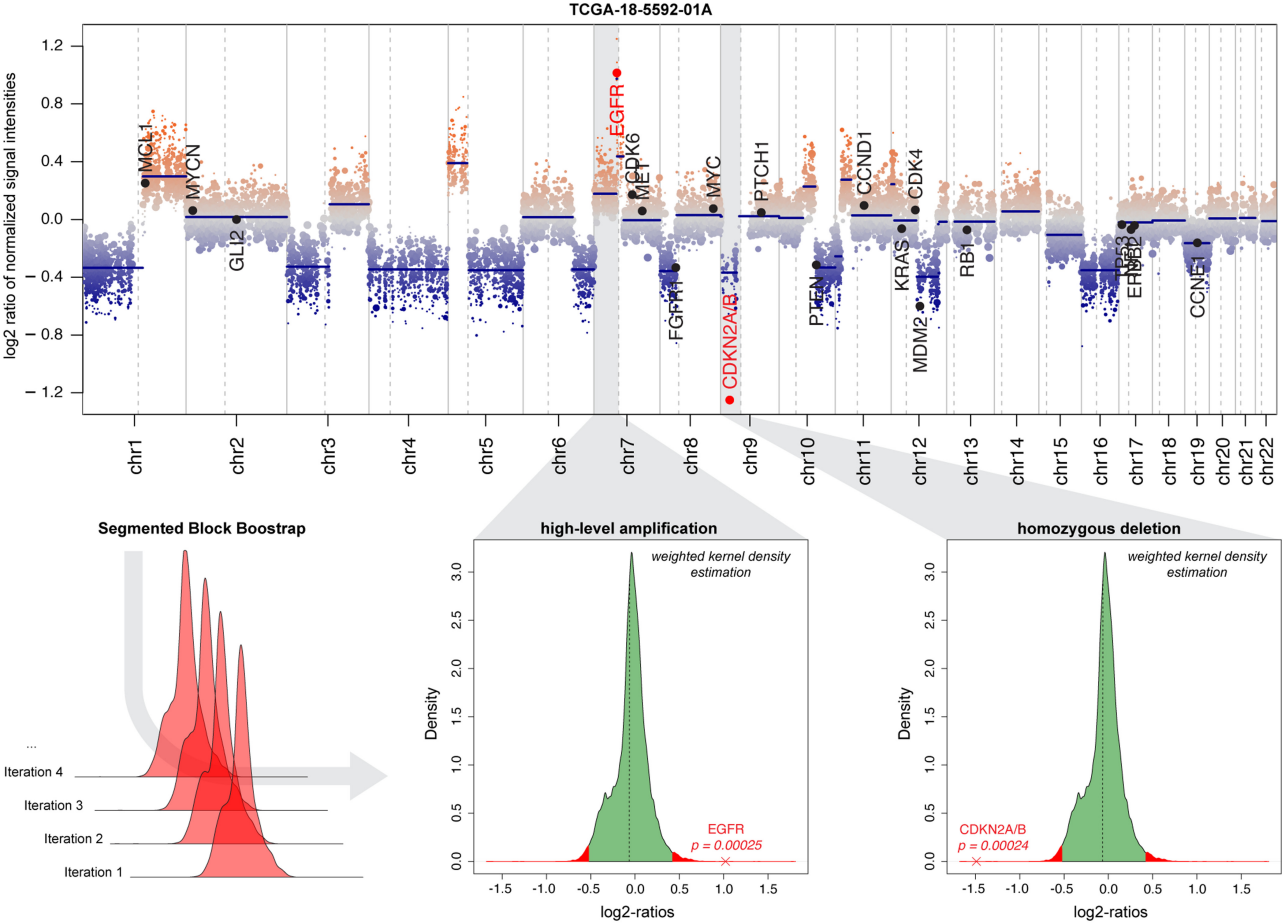
Detection of high-level alterations. Genome plot shows CNVs across a single query sample from the TCGA LUSC cohort. Indicated regions harbor a high-level amplification of *EGFR* and a homozygous deletion of the *CDKN2A/B* locus. Density plots illustrate the segmented block bootstrapping approach which allows for the calculation of empirical *P*-values to assess the statistical significance of identified focal CNVs.

In the revised version of our tool, we implement segmented block bootstrapping [from the nullranges package (Mu *et al.* 2023)] to detect focal alterations. This method generates random bootstraps of the original dataset by sampling large blocks of bins to calculate empirical *P*-values (Fig. 4). After a sample-specific two-sided confidence interval is defined, we identify significant focal CNVs that affect user-defined genes-of-interest and a list of over 700 common onco- and tumor suppressor genes from the Cancer Gene Census (Tate *et al.* 2018). These genes are annotated in genome plots and summarized in text-based output files.

For validating the approach, we analyzed the TCGA low grade glioma (LGG) cohort comprising 239 samples with paired SNP array (Affymetrix SNP 6.0) and methylation array (Illumina 450k) data. We selected a cohort of 53 copy-number neutral reference samples from the cerebral hemispheres, the cerebellum, white matter and the tumor microenvironment (Supplementary Table S1). Using SNP array data as a reference, we were able to achieve a sensitivity of 60.9% (14/23) and a specificity of 98.6% (213/216) for the detection of homozygous deletions affecting *CDKN2A/B*. We observed a similar performance for the detection of homozygous deletions in *RB1* (sensitivity: 6/10 = 60%; specificity: 222/229 = 96.9%), outperforming the recently described conumee K_CN_ approach (Supplementary Fig. S6).

### 3.5 Extended plotting functionality

**Figure 5.**
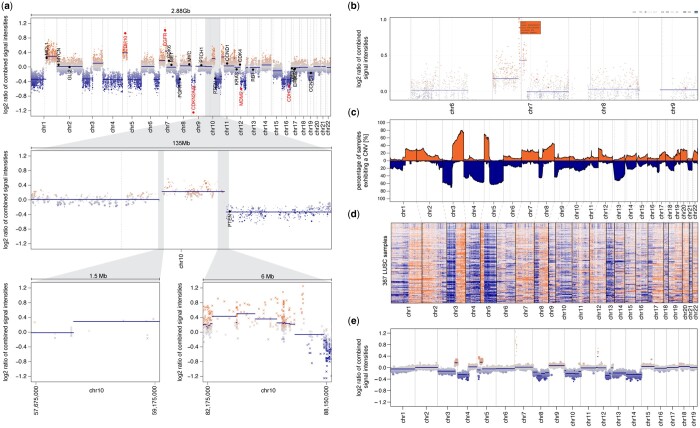
Overview of plotting functionality in conumee 2.0. (a) Genome plots show CNVs for a single query sample across the whole genome (top), chromosome 10 (middle), and two detail regions (bottom). (b) Interactive genome plot generated using the CNV.plotly function which can be used to identify genes within bins of interest. (c) Summary genome plot generated using the CNV.summaryplot function illustrates the percentage of samples exhibiting CNVs across the genome for the TCGA LUSC cohort. (d) Summary genome heatmap generated using the CNV.heatmap function shows bin-level intensities across the genome for the TCGA LUSC cohort (367 samples). (e) Genome plot showing a CNV profile of a mouse sample.

To enable its usage in clinical and research settings, conumee 2.0 supports a number of customizable parameters and user-friendly plotting functions to produce publication-grade illustrations of CNV results. The basic genome plot function produces CNV profiles of the whole genome, individual chromosomes, or other user-specified genomic regions (Fig. 5a). These plots show both segmentation results from the circular binary segmentation and indicate copy-number status of individual genes of interest. By default, these genes include 20 of the most common onco- and tumor suppressor genes. Genes can also be specified via a customizable annotation object. Similarly, an annotation object of polymorphic regions such as the human HLA gene locus, which should be excluded from the analysis, can also be defined. Both annotation objects provide versatility for different experimental contexts. To accelerate analytical workflows, we also implemented wrapper functions for the simultaneous analysis of multiple query samples.

In addition, conumee 2.0 supports new interactive plotting functions to facilitate the identification of genes within genomic regions of interest (Fig. 5b). The revised version also provides plotting functions to summarize CNV results across multiple query samples as a heatmap (Fig. 5c), or in a summary genome plot (Fig. 5d): The *y*-axis indicates the percentage of samples exhibiting a certain CNV at the genomic location on the *x*-axis, separated into gains and losses. Conumee 2.0 also provides plotting functionality for the analysis of mouse arrays (Fig. 5e).

## 4 Discussion

In this work, we present our R package “conumee 2.0,” a substantially improved version of “conumee.” We show that it is possible to infer accurate CNV profiles from DNA methylation microarrays, which is especially beneficial when sample material or resources are scarce, and a dedicated assay for CNV analysis (e.g. whole-genome sequencing or genotyping arrays) cannot be performed. We repurpose DNA methylation arrays for CNV analysis by using the sum of the unmethylated and methylated signal, followed by a series of normalization and processing steps. CNV analysis is of high relevance not only for cancer research, but also in clinical settings. Examples include the combined loss of chromosome 1p/19q as an essential diagnostic criterion for IDH-mutant oligodendroglioma, or homozygous deletions of *CDKN2A/B* as a grading criterion for IDH-mutant astrocytoma (WHO Classification of Tumours Editorial Board 2021). Accurate detection of CNVs is key for these applications.

Previous efforts to classify CNVs into gains and losses using fixed or dynamic log_2_-ratio thresholds provided low sensitivity, impeding their use in clinical settings (Kilaru *et al.* 2020). The block bootstrapping approach implemented in the revised version of conumee substantially improves the performance for detecting focal CNVs, thereby addressing a critical demand in clinical diagnostics and in research. Conumee 2.0 also provides functionality to annotate newly identified focal CNVs by overlapping results with extensive sets of described onco- and tumor suppressor genes, by providing interactive plotting functions, and by generating text-based output files for seamless integration with downstream tools such as GISTIC 2.0.

According to a recent publication by Gao *et al.* (2022), CNV inference from microarray data is subject to at least three sources of noise: Stochastic variability, the misinterpretation of germline CNVs as somatic ones, and systematic noise due to experimental conditions. A major source of technical noise in DNA methylation arrays results from the whole genome amplification step that is performed after bisulfite conversion during experimental processing (Bundo *et al.* 2012). Due to differences in experimental conditions (e.g. technical equipment), input material (e.g. amount and integrity of genomic DNA), sequence-specific effects (e.g. GC-bias), and stochastic effects (e.g. random primer binding during whole genome amplification), genomic DNA is not uniformly amplified, thereby impeding CNV analysis. Another challenge for CNV analysis from DNA methylation arrays lies in the probe design: Probe density varies across the genome, with most probes being located within CpG islands near gene promoters. We recommend choosing other sequencing-based methods to assess focal intergenic alterations like short insertions and deletions that may not be sufficiently covered by the array. Also, Illumina DNA methylation arrays comprise two different probe types (type I and type II) that show considerable differences in measured intensities, further complicating CNV analysis (Bibikova *et al.* 2011). To address these challenges, conumee 2.0 implements an optimized tangent normalization method, applies an adaptable genomic binning heuristic, and performs weighted CNV segmentation that takes into account the variability of probe intensities in control samples. We recommend excluding samples with a noise parameter >0.6 from analysis, although broad CNVs may be detected. We validate the performance of conumee 2.0 by comparing to CNV segmentation results generated from genotyping arrays, showing overall high agreement between both methods and highlighting the suitability of DNA methylation arrays for CNV analysis.

For tangent normalization, we recommend using a set of at least 16 copy-number neutral control samples, ideally generated using the same experimental pipelines, and from a related biological tissue (e.g. normal human brain tissues as a control for brain tumors samples). However, we have achieved good results even with control samples that were unrelated to the query cohort. It is important that the quality of control samples spans the range of qualities that can be observed in query samples (i.e. control samples that contain a certain technical artifact might be important for normalizing query samples that contain the same artifact). Also, it is advisable to include control samples generated from fresh-frozen and formalin-fixed, paraffin-embedded (FFPE) material if the query cohort contains samples from the same material.

For merging of individual probes into genomic bins, a minimum number of probes per bin and a minimum size per bin are required. Default parameters represent a compromise between reducing technical noise and higher genomic resolution that has resulted in good results in most cases. These parameters can be adapted to match the needs of the analysis. Depending on the number of genomic probes included in the array type (Illumina 450k, EPIC, EPICv2, or mouse array), a different number of genomic bins are formed using default parameters.

Our package conumee 2.0 enables enhanced CNV analysis from Illumina DNA methylation array data of human (including the new EPICv2 array) and mouse samples. We make use of the high genomic coverage of DNA methylation arrays to generate detailed CNV profiles using a set of specialized algorithms. Customizable annotation objects facilitate the analysis of individual genes-of-interest, and high-level alterations are detected de-novo using a novel segmented block bootstrapping approach. We assess the performance of our approach using publicly available datasets that comprise both DNA methylation and SNP array data from matching samples. Conumee 2.0 also introduces new summary functions to analyze sets of query samples and generates text-based output files for downstream-processing using popular public tools (e.g. GISTIC 2.0). DNA methylation array profiling is frequently performed in cancer research and clinical settings, and detailed CNV profiling from DNA methylation arrays adds an important layer of information.

## Supplementary Material

btae029_Supplementary_Data

## Data Availability

The raw IDAT files for the TCGA LUSC cohort (level 1 data) were downloaded from the TCGA public repository. The segmentation files from the Affymetrix SNP6 arrays (TCGA level 3 data, downloaded in September 2022) were downloaded from the Broad Institute’s Firehose Genome Data Analysis Center (data analysis version: 2016_01_28). The IDAT files for the TCGA LGG cohort were downloaded from the TCGA public repository (level 1 data). The 53 reference samples that were used for the LGG cohort were downloaded from GEO (GSE109381, Supplementary Table S1). The copy-number states for *CDKN2A/B* and *RB1* inferred from Affymetrix SNP6 arrays were downloaded using the TCGAbiolinks package (January 2023) (Colaprico *et al.* 2015).

## Availability and requirements

Project name: Conumee 2.0.

Project home page: https://github.com/hovestadtlab/conumee2.

Operating system: Platform independent.

Programming language: R (≥ 4.1).

Dependencies: minfi, IlluminaHumanMethylation450kmanifest, IlluminaHumanMethylation450kanno.ilmn12.hg19, IlluminaHumanMethylationEPICmanifest, IlluminaHumanMethylationEPICanno.ilm10b4.hg19, RnBeads, illuminaio, nullranges, plotly.

Hardware requirements: Linux or Mac OS, 8GB of RAM, >1.6 GHz CPU.

License: GPL-2.0 open-source license.

Any restrictions to use by non-academics: no restrictions.
