# Supplementary material for: Conumee 2.0: enhanced copy-number variation analysis from DNA methylation arrays for humans and mice
Source: Bioinformatics. 2024 Jan 19;40(2):btae029. doi: 10.1093/bioinformatics/btae029 (PMC10868300; doi:10.1093/bioinformatics/btae029)
Supplement: btae029_Supplementary_Data [file btae029_supplementary_data.zip › Daenekas_Conumee2_SupplementaryFigures.Final.pdf]

Supplementary Material

Table of Contents

Supplementary Figure 1: Contributions of reference samples ……………………………………..……. 2

Supplementary Figure 2: Comparison of observed and predicted log2-transformed signal intensities …. 3

Supplementary Figure 3: Benchmarking of CNV segmentations …..…………..………………………. 3

Supplementary Figure 4: Evaluation of CNV threshold values ………………...…..…………………… 4

Supplementary Figure 5: Evaluation of CNV calls ………………………………………........……. 4

Supplementary Figure 6: Benchmarking of focal alteration detection ……………………..…...……. 5

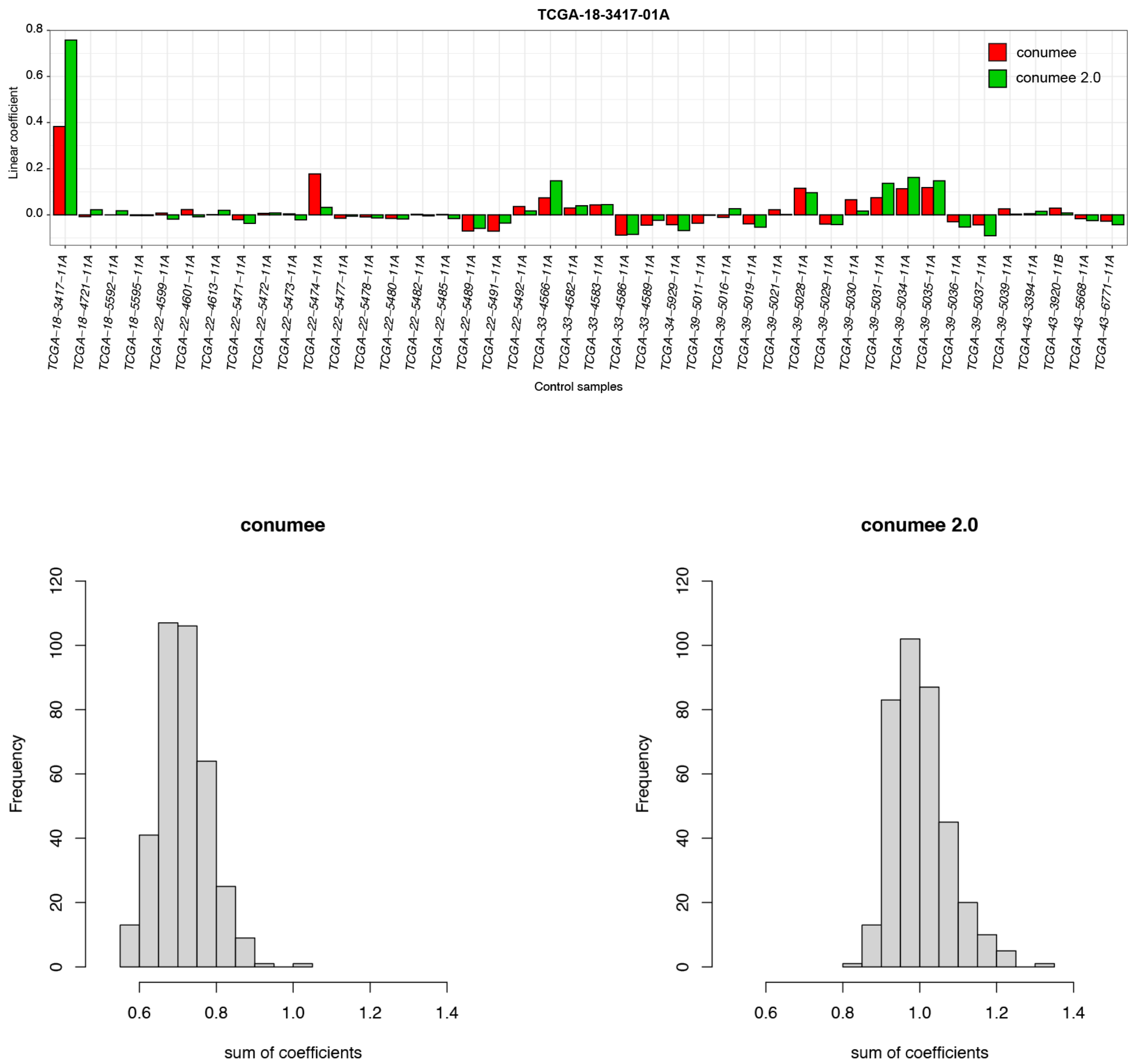

Suppl. Fig. 1 - Contributions of reference samples. The absolute values from the linear coefficients of every reference sample (with their sums adding up to 1) are shown (top). One sample from the TCGA LUSC cohort was used for this plot. The sums of the linear coefficients follow a unimodal distribution (bottom). The TCGA LUSC cohort (n=367) was used for the plots.

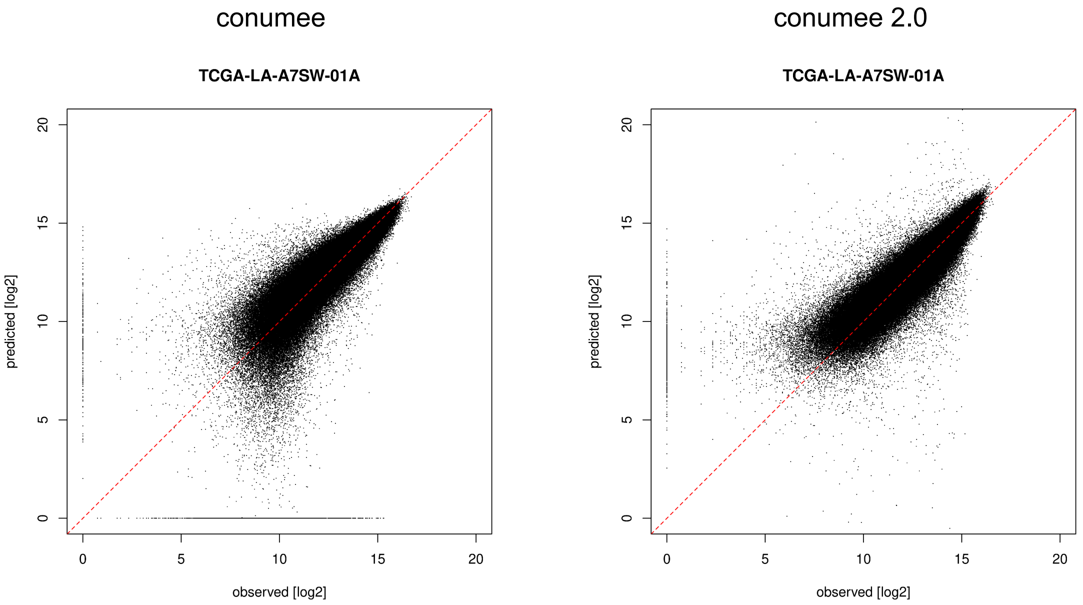

Suppl. Fig. 2 - Comparison of observed and predicted log2-transformed signal intensities. We compare the Tangent Normalization between conumee (left) and conumee 2.0 (right). In the original approach, we fit a linear model of reference samples to untransformed added signal intensities. In the update, we fit this model to log2-transformed values.

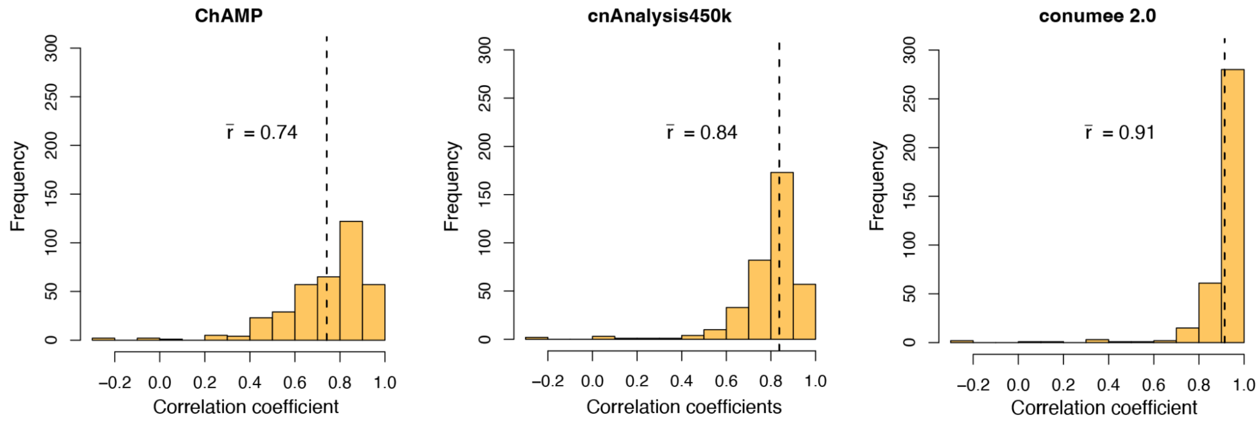

Suppl. Fig. 3 - Benchmarking of CNV segmentations. We compare the segmentation results from conumee 2.0 to the segmentation results from ChAMP and cnAnalysis450k. We calculated the log2-ratio on gene-level and determined the correlation coefficients between matching samples in the methylation and SNP array cohort. The mean Pearson correlation coefficient is shown. The TCGA LUSC cohort was used for this comparison (n = 367).

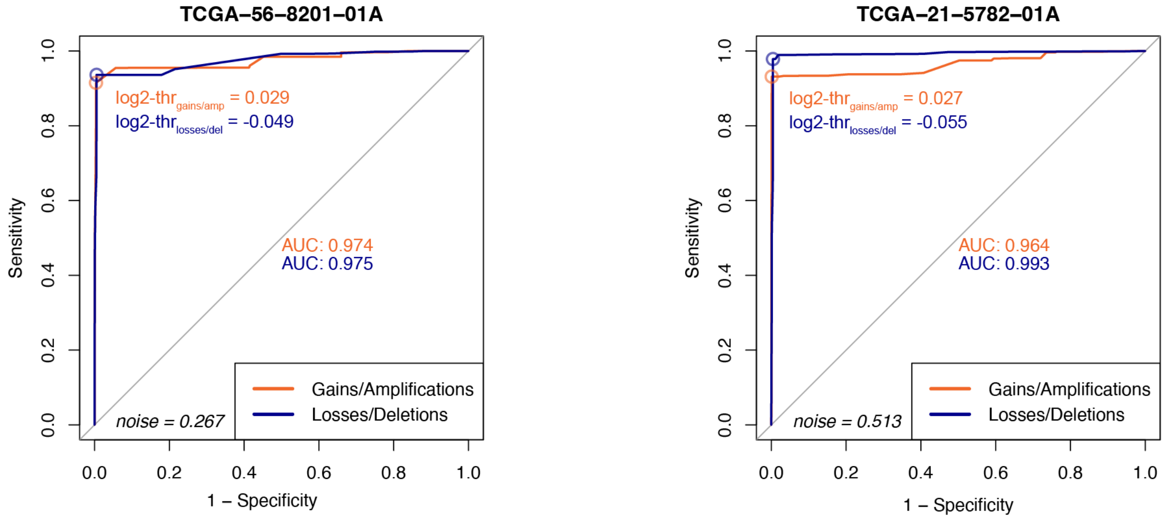

Suppl. Fig. 4 - Evaluation of CNV threshold values. Receiver operating characteristic (ROC) curves illustrating the detection of gains/amplifications (orange) and losses/deletions (blue) for different log2-ratio thresholds. Shown are two samples with varying noise parameters (left: low noise, right: high noise). Optimal log2-ratio thresholds are different for every sample using Youden's J statistic. Based on this analysis, universal thresholds of -0.04 and 0.04 were selected as appropriate values for losses and gains, respectively.

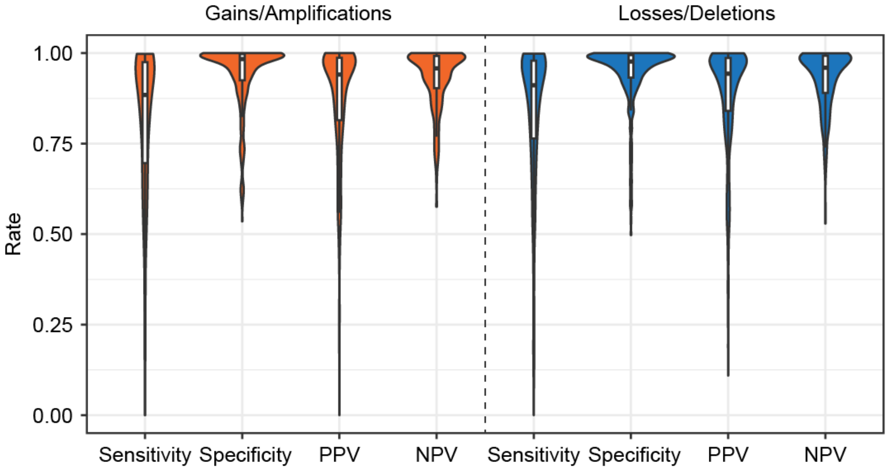

Suppl. Fig. 5 - Evaluation of CNV calls. Violin plots show the consistency of conumee-derived CNV calls with results obtained from SNP arrays across samples from the TCGA LUSC cohort (sensitivity, specificity, positive predictive value, negative predictive value are shown). Thresholds of -0.04 and 0.04 are used. Gains/amplifications, and losses/deletions are analyzed separately.

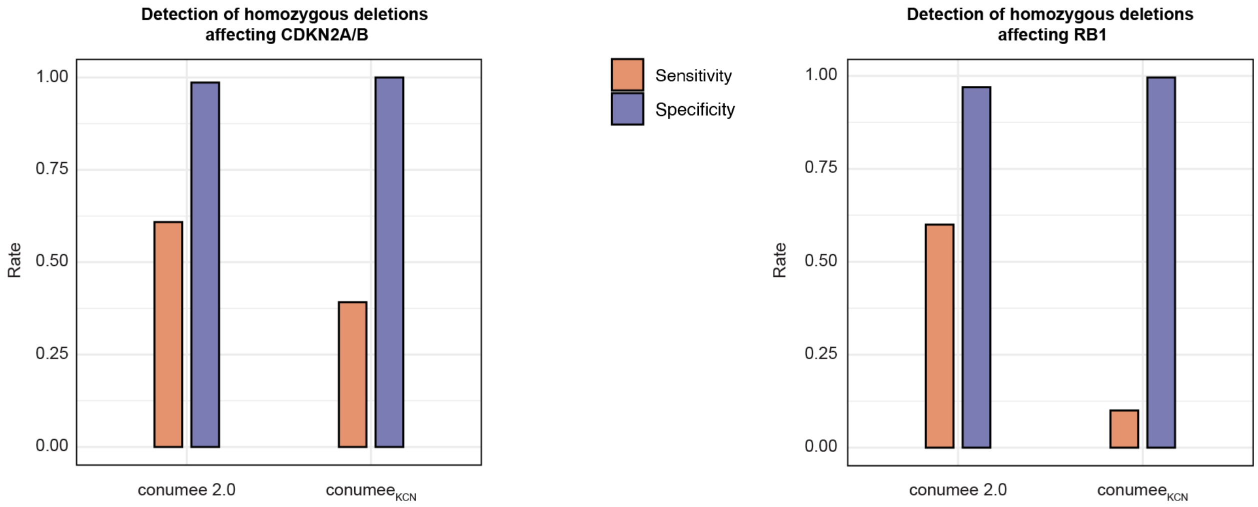

Suppl. Fig. 6 - Benchmarking of focal alteration detection. We compare conumee 2.0 that implements Segmented Block Bootstrapping for the detection of focal CNVs to conumee K_{CN}'s dynamic thresholding approach. The TCGA LGG cohort was used for this comparison (n = 239).
